# Magnetic resonance and computed tomography imaging of a carotid body tumor in a dog

**DOI:** 10.1186/1751-0147-54-24

**Published:** 2012-04-16

**Authors:** Kaatje Kromhout, Ingrid Gielen, Hilde EV De Cock, Kristof Van Dyck, Henri van Bree

**Affiliations:** 1 Faculty of Veterinary Medicine, Department of Medical Imaging of domestic animals and Orthopedics of small animals, Ghent University, Salisburylaan 133, 9820, Merelbeke, Belgium; 2 Medvet - Veterinary Pathology Services, Emile Vloorstraat 9, 2020, Antwerpen, Belgium; 3 Dierenartspraktijk Van Dyck, Merksemsebaan 138, 2110, Wijnegem, Belgium

**Keywords:** CT, MRI, Carotid body tumor, Paraganglioma, Chemodectoma

## Abstract

A 5-year-old castrated male Labrador Retriever was presented to a referring veterinarian for a swelling in the neck region. Based on the results of histopathology, a carotid body tumor, was diagnosed. The dog was referred to a medical imaging unit for further staging and follow up. This report describes the magnetic resonance (MR) and computed tomographic (CT) appearance of a carotid body tumor.

## Background

Chemodectomas or paragangliomas are tumors of chemoreceptor cells. In dogs they originate most commonly from the aortic or carotid bodies. Carotid body tumors appear less frequent [1] and are usually more malignant than aortic body tumors [2]. These tumors are usually non-functional. Generally, they cause clinical symptoms late in the course of the disease, as they exert a space occupying mass effect on the surrounding structures. Carotid body tumors tend to splay the carotid bifurcation as they enlarge [3], and treatment is difficult. This report describes the magnetic resonance (MR) and computed tomographic (CT) appearance of a histological confirmed carotid body tumor.

### Case presentation

A 5-year-old castrated male Labrador Retriever was presented to a referring veterinarian for a swelling in the neck region. On physical examination a hard, painless, not retractable spherical mass, the size of a golf ball (+/ 55cm), was palpated just caudally of the left mandibula, in the region of the mandibular lymph node. A fine needle aspiration was performed and the dog was placed on antibiotics pending the outcome. As cytology results were inconclusive, mainly consisting of necrotic cell debris, antibiotic therapy was stopped and it was decided to remove the mass for histopathological examination. During surgical exploration a second, more oval, 154cm mass, located caudally from the first one, was noticed. This caudal mass was left in place and no biopsy samples were taken from it.

**Figure 1:**
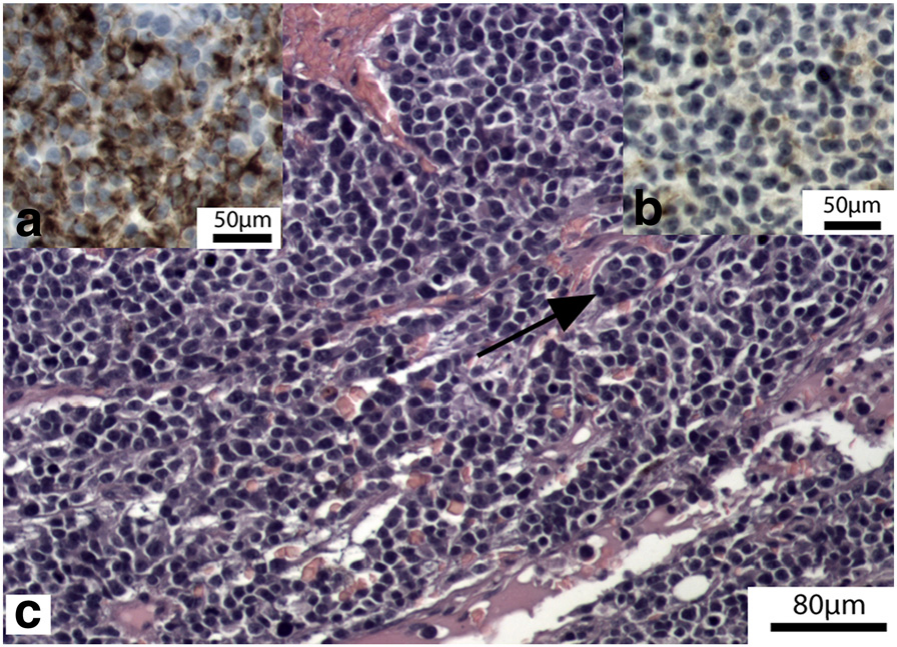
**Lymph node metastasis of the carotid body tumor in a lymph node (c), composed of small nests of large pleiomorphic polygonal or oval cells (arrow) with a large round nucleus with coarse chromatin and inconspicuous nucleoli, and a variable amount of granular, slightly basophilic cytoplasm (H&H stain; bar=80m).** Also notice the mild cytoplasmic staining with anti-chromogranin A antibodies (inset right, b, immunohistochemical stain with anti-chromogranine A, bar=50m) and strong cytoplasmic staining with anti-vimentin antibodies (inset left, a, immunohistochemical stain; anti-vimentin, bar=50m)

On histopathological examination, the first cranial spherical mass noticed, turned out to consist of a reactive lymph node with metastasis of an anaplastic carcinoma. The metastatis consisted of small nests composed of large pleiomorphic polygonal or oval cells with a large round nucleus with coarse chromatin and inconspicuous nucleoli, and a variable amount of granular, slightly basophilic cytoplasm. Anisocytosis, anisokaryosis and karyomegaly was prominent (Figure 1). Mitotic figures were numerous, ranging from 58 mitotic figures/high power field (HPF). The neoplastic nests were surrounded by a fine fibrovascular stroma. Randomly distributed, there were small foci with necrosis. Immunohistochemical evaluation with a panel of commercially available mouse and rabbit anti-human primary monoclonal antibodies (DAKO Corp.; Heverlee, Belgium) remained negative for pan cytokeratin (clone AE1/AE3), Melan A (clone A103), CD20 (polyclonal), CD3 (polyclonal) and a mouse monoclonal anti canine CD18 (clone CA16.3C10; P. Moore; University of California, Davis, USA) antibody remained negative. Positive immunohistochemical staining was detected with human mouse monoclonal anti-vimentin (V9) and Chromogranin A (polyclonal) antibodies (Figure 1). Based on morphology of the tumor and immunoreactivity the diagnosis of neuroendocrine tumor, more specific paraganglioma was made. Because of the anatomical location, a paraganglioma of the carotid body was suspected.

### Diagnostic imaging

To rule out additional metastasis survey thorax radiographs (lateral and ventrodorsal) were performed. No radiographic abnormalities were observed. Subsequently 3weeks after exploratory surgery the dog was send to the Department of Veterinary Medical Imaging and Small Animal Orthopaedics for a MR scan of the mandibular and cervical region and a CT scan of the thorax. The CT procedure was performed with a multi-slice helical CT scanner (GE Lightspeed QX/I; General Electric Co., Milwaukee, MI, USA) with the patient under general anaesthesia and in ventral recumbency. Images were obtained before and immediately after administration of 2ml/kg intravenous contrast medium (Ultravist 300; N.V. Shering S.A.). Both pre- and post contrast images revealed no abnormalities at the level of the thorax.

**Figure 2:**
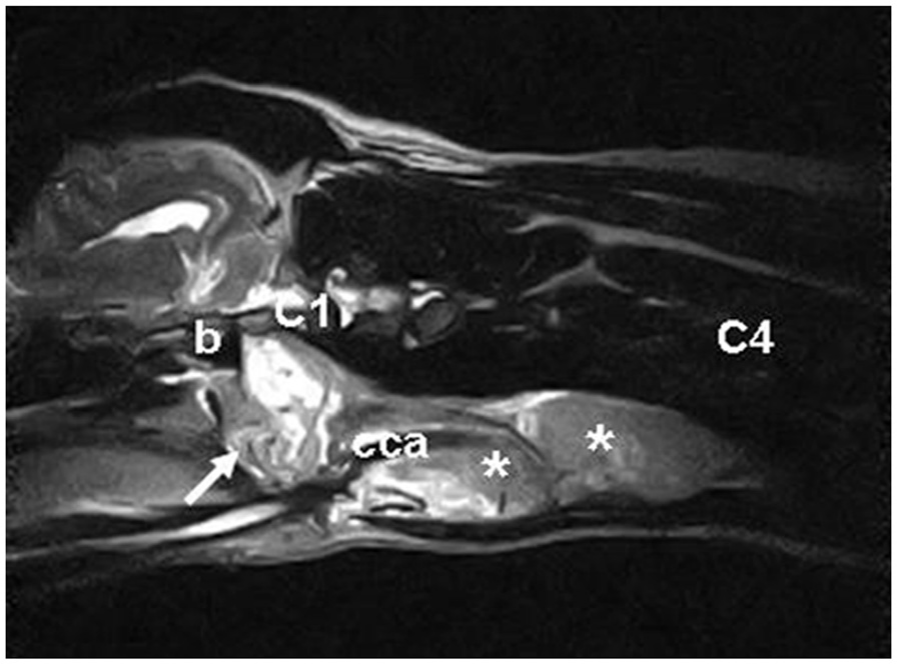
**Sagittal T2W SE image of the left cervical region.** A heterogenous, hyperintense mass (carotid body tumor) (arrow) with multiple signal voids is visible caudal from the bulla (b) to the caudal part of the atlas (C1). A lobulated, elongated more homogeneous mass (medial retropharyngeal lymph node) (asterisk) is visible streching out to the caudal part of C4. The CCA (cca) is visible laterally from this mass.

**Figure 3:**
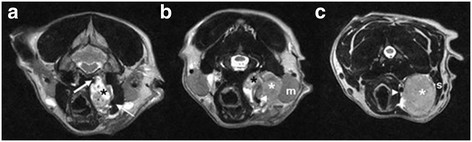
**Transverse T2W SE images at the level of the caudal part of the bulla (a), cranial part of the atlas (b) and the mid part of the axis (c).** A heterogenous, hyperintense mass (carotid body tumor) (black asterisk) is visible on the left side, with multiple signal voids of vessels. The mass splays the ICA more medially (large arrow) and the ECA more laterally (small arrow). Laterally a homogeneous, hyperintense encapsulated mass (medial retropharyngeal lymph node) (white asterisk) is visible. The mass is located between the CCA (arrowhead) and the mandibular gland (m) (panel b) and more caudally between the CCA and the m. sternocephalicus (s).

To identify the extent of the lesion, a MRI (0.2 Tesla; Airis Mate; Hitachi Medical Corporation, Japan) examination of the mandibular and cervical region was performed with the dog in dorsal recumbency and the cervical region in a human neck coil. The MRI procedure was performed immediately after the CT-scan, with the dog still under general anaesthesia. Images were obtained in three planes (sagittal, transverse and dorsal). Sequences included T1-weighted (T1W), T2-weighted (T2W) spin echo (SE) and short T1 inversion recovery (STIR). T1W SE were also aquired after intravenous paramagnetic contrast medium (0.3ml/kg; Magnevist; Bayer HealthCare Pharmaceuticals Inc.) injection. On the sagittal T2W SE images (Figure 2) an elongated, heterogeneous, hyperintense mass (14.5cm length) was visible parasagittal left from the trachea. This mass contained two parts: a heterogeneous, hyperintense, not well defined part, extending from the caudal part of the left bulla to the caudal part of the atlas (3.1cm length) and an elongated more homogeneous, hyperintense, well defined lobulated (11.4cm length) part with hyperintense regions, stretching out to the caudal part of the fourth cervical vertebrae. On T1W SE images the mass appeared heterogeneous, moderate mixed iso-, hypo- to hyperintense. On the transverse T2W SE images (Figure 3) the cranial part of the mass was visible at the medioventral border of the left bulla on the level of the rostral border of the cerebellum. It appeared as a strong heterogeneous, hyperintense, well defined, encapsulated mass with multiple round hypointense regions. The mass stretched out dorsally between the oropharynx and the m. digastricus and splayed the internal carotid artery (ICA) more medially and the external carotid artery (ECA) ventrolateral. These arteries appeared as signal voids besides the mass. The mass deviated the oro- and laryngopharynx and the m. longus capitis to the right side. At the level of the laryngopharynx the second part of the mass appeared between the mandibular salivary gland and the first more rostral part of the mass. It appeared as a more homogeneous, hyperintense, well defined, encapsulated mass. More caudally it is located lateral from the common carotid artery (CCA), medial from the deviated m. sternocephalis, m. sternomastoideus and m. cleidomastoideus and ventral from the m. longus capitis which is deviated to the right. Due to the localisation and the appearance this lobulated part is described as a severe enlarged and lobulated medial retropharyngeal lymph node. On the dorsal STIR- and T1W SE-images the extent and the localisation of the full mass compared to the surrounding structures is visible. On the post-contrast T1W SE images a homogenous intense contrast uptake is visible within the cranial part of the mass and a homogeneous mild contrast uptake is visible within the caudal part of the mass with a uptake in the surrounding capsule.

### Treatment and follow up

**Figure 4:**
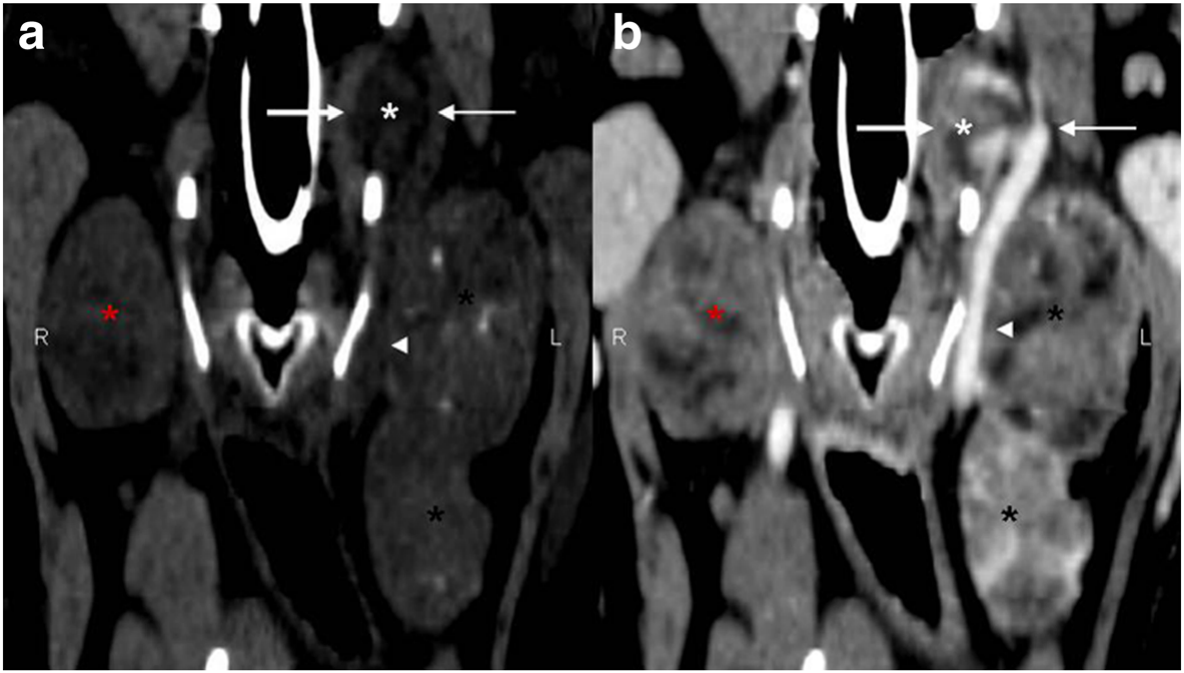
**Dorsal reformatted CT images of the cervical region.** Images are displayed in a soft tissue window. The pre-contrast image (a) shows a well defined soft-tissue mass (carotid body tumor) (white asterisk) splaying the ICA medially (large arrow) and the ECA laterally (small arrow). Also a more lobulated, elongated, encapsulated soft- tissue mass (medial retropharyngeal lymph node) (black asterisk) with areas of calcification is visible on the left side, laterally from the CCA (arrowhead). On the right side a singular soft-tissue mass (medial retropharyngeal lymph node) (red asterisk) is visible. On the post-contrast image (b) the mass shows a heterogenous intense contrast enhancement with areas of low density (necrosis). Both the elongated mass on the left side and the singular mass on the right side have a contrast uptaking capsula. The splaying of the ICA and ECA by the mass is clearly visible.

**Figure 5:**
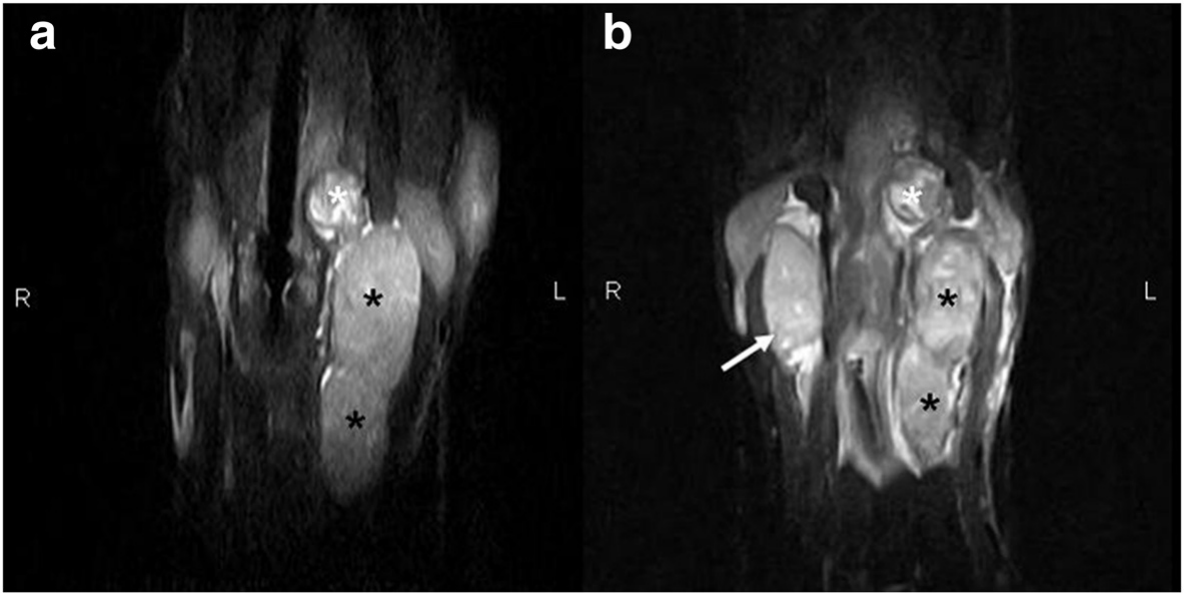
**Dorsal STIR images of the cervical region during the first examination (a) and the follow up examination (b).** The primary heterogenous mass (carotid body tumor) (white asterisk) and the secondary lobulated, homogeneous mass (medial retropharyngeal lymph node) (black asterisk) appear to be decreased in size (panel a compared to panel b). A secondary, homogeneous, encapsulated medial retropharyngeal lymph node (arrow) is visible on the right side (panel b).

Because of the relationship of the tumor with the carotid vessels and its extent there was decided not to remove the tumor but to start radiation therapy. The treatment was tolerated well and the tumor decreased in volume. Two months after treatment a follow up MR-examination was performed. An additional CT-examination of the thorax and abdomen was recommended because on recent physical examination an enlarged cervicalis superficialis lymph node was observed. The dog also had difficulties with urination, and abdominal ultrasonography was performed. On ultrasound, the wall of the urinary bladder was uniformly thickened and hyper- echoic consisted with a cystitis. No masses were detected. The CT-scan revealed lymphadenopathy of several lymph nodes (cervicalis superficialis, mediastinales, renalis, hepaticus, mesenterici craniales and inguinalis superficialis), a heterogenous hyperdense enlarged right pancreas lobe and abnormal densities in the subpleural region of the left caudal lung lobe. On the CT images of the neck region (Figure 4) the mass was visible on the left side as a heterogeneous, hypodense, well-defined mass with regions of mineralization and necrosis. On the right side a severe enlarged medial retropharyngeal and mandibular lymph node was present. On post-contrast images a heterogeneous contrast uptake was visible in the mass and the lymph nodes. On the MR images the mass on the left side was still visible but was decreased in size (13.2cm length) (Figure 5). On the right side a homogeneous, hyperintense, encapsulated, enlarged medial retropharyngeal lymph node was visible (5.9cm length) and a homogeneous hyperintense enlarged mandibular lymph node was visible.

### Pathological examination

**Figure 6:**
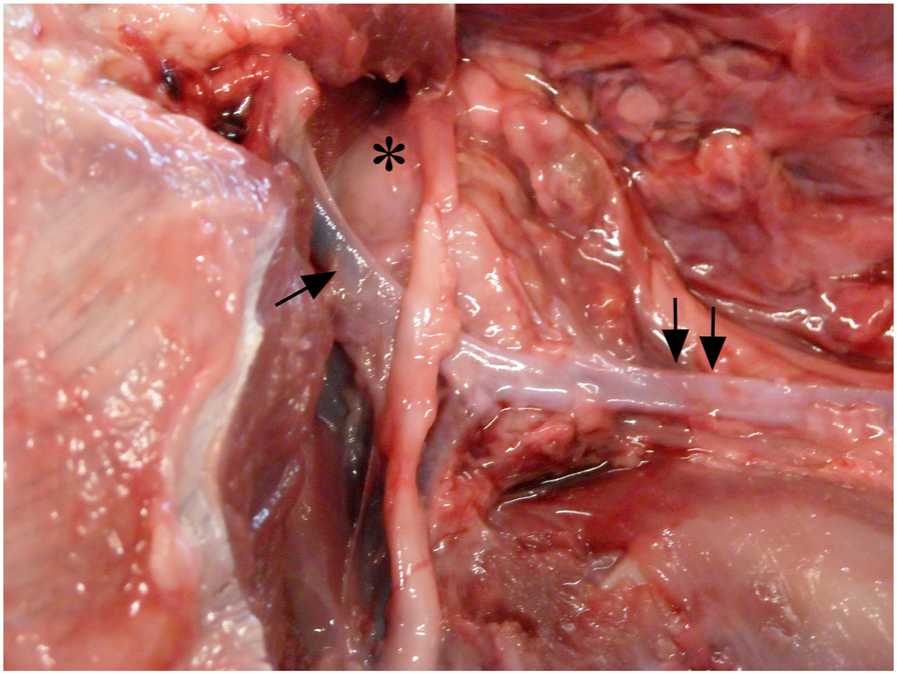
**Postmortem image of the left cranial cervical region.** The carotid body tumor (asterisk) is localized just craniomedial of the ECA (arrow). CCA is visible (two arrows).

Because of the poor prognosis, fast progressing disease and general discomfort it was decided to euthanize the dog. On necropsy (Figure 6) a 9.04.03.0cm and 7.04.04.0cm soft, multinodular mass respectively on the left and right neck region just caudal of the mandibula was found. The masses were gray on cut section with large green, hemorrhagic necrotic, sharply circumscribed areas. On the left side, cranioventrally of the largest mass, there was a second spherical mass, 2.5cm diameter. Most likely metastasis were found just cranially of the left thoracic inlet which connected with a large metastasis extending in de cranial mediastinum, and in the right ventricle of the heart. In the abdomen a metastatic, multinodular mass 11.09.04.0cm was located in the mesentery and incorporated several mesenterial and hepatic lymph nodes as well as the pancreas. In addition a metastasis was found cranial of the left kidney (renal lymph node).

## Discussion

To our knowledge this is the first report of the MRI and the CT appearance of a confirmed carotid body tumor in a dog. The most important differential diagnoses for cranial cervical masses are abcesses, thyroid hyperplastic or neoplastic nodules, enlarged retropharyngeal lymph nodes, salivary glands inflammation or neoplasias. Neuroendocrine tumors, although generally rare, should be considered as well.

A carotid body tumor is a type of chemodectoma or paraganglioma. These are tumors of the chemoreceptor organs which detect changes in arterial blood oxygen, carbon dioxide and pH. The carotid glomus and aortic body are the most common sides for development of chemodectomas in dogs [1]. The site of origin can also involve the tympanic cavity and inferior vagal ganglion [4]. The carotid body is located dorsal from the bifurcation of the common carotid artery. Carotid body tumors appear less frequent than aortic body tumors in dogs [1] and are more malignant [2]. There is a predisposition for Boxers and other brachycephalic dogs [4]. Clinical signs are usually caused by a mass effect of the growing neoplasia on the surrounding structures and by local invasion [5]. They tend to interfere with swallowing, cause dyspnoea and circulatory disturbances. Diagnosis in dogs is possible via imaging techniques such as ultrasonography [6], computed tomography [7] and histological diagnosis. The diagnosis of a neuroendocrine tumor in this case is based on the immunohistochemical expression of chromogranin A and vimentin [3]. Morphology of the neoplastic cells was suggestive for a carcinoma and more specific of neuroendocrine origin. However because of the high degree of pleiomorphism definitive diagnosis was only possible after immunohistochemical characterization.

In human medicine conventional ultrasound, color Doppler ultrasound, carotid conventional angiography (CA), axial tomography, magnetic resonance and magnetic resonance angiography, and computed tomographic angiography (CTA) are used to diagnose carotid body tumors [8]. On MRI carotid body tumors are heterogeneous iso- to hyperintense on T1W SE-images and heterogeneous hyperintense on T2W SE-images [9-11]. Regions of low signal intensity (signal voids or flow voids) appear in the mass due to the presence of high flow of vessels. This gives a salt-and-pepper heterogenity on T2W SE images, which is characteristic for paragangliomas, [10-12] but can also appear in other hypervascular lesions such as thyroid carcinomas [11]. The salt components are the high-signal regions which correspond with slow flow or hemorrhage and the pepper components are the multiple signal voids of vessels on both T1W- as T2W SE images. Post-contrast paragangliomas show typical homogeneous and intense enhancement because of their hypervascularity [12]. These findings are consistent with the findings seen on the images of our patient. On CT images, a carotid body tumor manifests as a well-defined soft-tissue mass within the carotid space with a homogeneous intense contrast enhancement. Large tumors are frequently inhomogeneous with areas of necrosis and hemorrhages [12]. These findings are also visible on our images and are similar to previous reports in dogs [7].

Metastases of carotid body tumors are present in approximately 30% [13] of the reported cases and tend to spread to regional lymph nodes, liver, lung, pancreas, kidneys and bones [2,14-16]. There is reported in humans that these tumors, although slow growing, are capable of rapid enlargement and metastasis at any stage of their evolution. This is consistent with our findings, where metastasis in the lymph nodes and pancreas were detected.

MRI, CT and other imaging techniques, such as RX or echo, are very usefull to determine the extent of the disease. A full clinical workup is essential for further planning and prognosis of the disease. The preferred treatment for carotid body tumors is surgical removal. MR imaging is the method of choice for surgical planning of the tumors [8,9]. In humans there is a surgical classification system to assess the resectability of carotid body tumors. Tumors are classified in three groups according to Shamblin et al. [17] and are based on the tumor size. Group I are small tumors which could easily be resected from the vessels, group II includes tumors that are intimately associated and compress carotid vessels, but that could be resected with careful subadventitial dissection and group III consists of tumors that are large and require total resection of the external and/or internal carotid artery. Cross-sectional imaging such as MR, because of the better soft tissue detail and higher contrast resolution than CT, can accurately predict the group and is essential for decrease of the perioperative mortality rate. Dogs with Shamblin group III tumors have a higher risk for perioperative death [2]. Judging from our CT and MR images the tumor of this dog belongs to Shamblin group II. If surgery is not possible, radiotherapy is recommended. Radiation therapy as a primary treatment, has not been described in humans and in animals. Radiation therapy is used for local control of the tumor, to eradicate microscopic disease and eventually prevent further spreading. Radiotherapy is helpful in cases with unresectable lesions, in high-risk patients and as additional treatment for incompletely excised tumors or metastases [18].

## Conclusions

Due to the specific localisation and high vascularity of the carotid body tumor, combined with the specific imaging characteristics, CT and especially MRI can be used as a primary diagnostic imaging technique and as a tool for presurgical planning and postsurgical assessment of dogs.

## Consent

Written informed consent was obtained from the owner for publication of this report and any accompanying images.

## Abbreviations

CCA = Common Carotid Artery; CT = Computer Tomography; ECA = External Carotid Artery; ICA = Internal Carotid Artery; MRI = Magnetic Resonance Imaging; STIR = Short T1 Inversion Recovery; T1W SE = T1-weighted Spin Echo; T2W SE = T2-weighted Spin Echo.

## Competing interests

The authors declare that they have no competing interests.
